# Application of noncontact sensors for cardiopulmonary physiology and body weight monitoring at home: A narrative review

**DOI:** 10.1097/MD.0000000000039607

**Published:** 2024-09-06

**Authors:** Yoo Jin Choo, Jun Sung Moon, Gun Woo Lee, Wook-Tae Park, Heeyeon Won, Min Cheol Chang

**Affiliations:** a Department of Physical Medicine and Rehabilitation, College of Medicine, Yeungnam University, Daegu, Republic of Korea; b Division of Endocrinology and Metabolism, Department of Internal Medicine, College of Medicine, Yeungnam University, Daegu, Republic of Korea; c Department of Orthopaedic Surgery, College of Medicine, Yeungnam University, Daegu, Republic of Korea; d Regional Leading Research Center on Development of Multimodal Untact Sensing for Life-Logging, Yeungnam University Industry-Academic Cooperation Foundation, Gyeongsan-si, Gyeongsangbuk-do, Republic of Korea.

**Keywords:** health status, home, monitoring, noncontact, sensor

## Abstract

Monitoring health status at home has garnered increasing interest. Therefore, this study investigated the potential feasibility of using noncontact sensors in actual home settings. We searched PubMed for relevant studies published until February 19, 2024, using the keywords “home-based,” “home,” “monitoring,” “sensor,” and “noncontact.” The studies included in this review involved the installation of noncontact sensors in actual home settings and the evaluation of their performance for health status monitoring. Among the 3 included studies, 2 monitored respiratory status during sleep and 1 monitored body weight and cardiopulmonary physiology. Measurements such as heart rate, respiratory rate, and body weight obtained with noncontact sensors were compared with the results obtained from polysomnography, polygraphy, and commercial scales. All included studies demonstrated that noncontact sensors produced results comparable to those of standard measurement tools, confirming their excellent capability for biometric measurements. Overall, noncontact sensors have sufficient potential for monitoring health status at home.

## 1. Introduction

Recently, monitoring health status at home has attracted increasing interest and demand. People are highly interested in managing their health and adopting better lifestyles.^[1]^ Consequently, health monitoring at home is recognized as an important tool for managing and improving individual health.^[2,3]^ The growing older population has also contributed to the increased interest in home health monitoring.^[4]^ Older individuals often feel a greater need for medical services, and monitoring their health at home is a convenient and safe option.^[4,5]^ In addition, in situations with limited access to medical services, such as with long distances from hospitals or mobility issues, home health monitoring can significantly assist in personal health management.^[6]^ Another reason is the high prevalence of infectious diseases. During outbreaks such as the Coronavirus disease 2019, people tend to spend a significant amount of time at home and avoid visiting medical facilities.^[7]^ Consequently, online health services and health monitoring devices have seen increased demand.^[8]^ Home health monitoring has several benefits. Individuals can manage their health in a comfortable environment without needing to visit hospitals, thus saving the associated time and cost.^[6,9]^ Moreover, regular health monitoring provides opportunities for the early detection and prevention of health issues and can aid in establishing personalized treatment and management plans.^[10,11]^ Therefore, monitoring health status at home is beneficial for maintaining health and preventing diseases.

So far, health monitoring at home has primarily involved wearable devices.^[12,13]^ However, patients experience discomfort while wearing such devices, and wearing them throughout the day presents practical hurdles, resulting in constraints on continuous health monitoring with these devices.^[14]^ Additionally, the accuracy of biometric measurements can be compromised by fluctuations in motion artifacts or foreign bodies, such as sweat or water.^[15,16]^ To address this issue, noncontact sensors that do not interfere with activities and do not require physical contact with the patient’s body have been gaining increasing attention. Noncontact sensors typically use energy, such as light, sound, or heat, to detect and measure properties such as the position, distance, temperature, and movement of the target object.^[17,18]^ With the advancement of sensing technology, the application of noncontact sensors in the medical field has been actively researched, and noncontact sensors have proven useful for measuring various types of biometric information, including body temperature, heart rate, and respiratory rate.^[19,20]^ The potential of utilizing noncontact sensors in home settings has been recently evaluated. In 2009, Zaffaroni et al^[21]^ reported the excellent monitoring performance of a noncontact home respiratory signal analysis device for sleep-disordered breathing. In 2020, Yoon et al^[22]^ reported that home-based noncontact sensors demonstrated high accuracy in estimating sleep stages (wakefulness, rapid eye movement, light sleep, and deep sleep) for managing sleep quality. Based on these previous studies, noncontact sensors can be effectively utilized to monitor the health status of patients at home.

In this study, we aimed to investigate the potential feasibility of using noncontact sensors in home environments by reviewing previous studies evaluating the performance of noncontact sensors for actual use in home settings.

## 2. Methods

### 2.1. Search strategy

Relevant studies published until February 19, 2024, were searched on PubMed using the following keywords: “home-based,” “home,” “monitoring,” “sensor,” and “noncontact.” The inclusion criteria for the studies were as follows: (1) research that monitored the health status of patients by installing noncontact sensors in their homes and (2) articles written in English. The exclusion criteria were as follows: (1) studies involving only healthy individuals, (2) studies using wearable devices as noncontact sensor devices for monitoring health status, and (3) technical development reports, reviews, letters to the editor, conference presentations, or other unidentified article types. This study confirms to SANRA guidelines and reports the required information accordingly (Appendix 1, Supplemental Digital Content, http://links.lww.com/MD/N533).

### 2.2. Methodological quality assessment

The methodological quality assessment of the included studies was performed using the quality assessment of diagnostic accuracy studies (QUADAS)-2 tool. The QUADAS-2 tool was developed to evaluate the risk of bias and applicability in diagnostic accuracy studies.^[23]^ The domains for assessing risk of bias in the QUADAS-2 tool were as follows: (1) patient selection, (2) index test, (3) reference standard, and (4) flow and timing. Two independent reviewers (Y.J.C. and M.C.C.) conducted the assessment, and any disagreements were resolved through discussion.

## 3. Results

### 3.1 . Selection of studies

**Figure 1.**
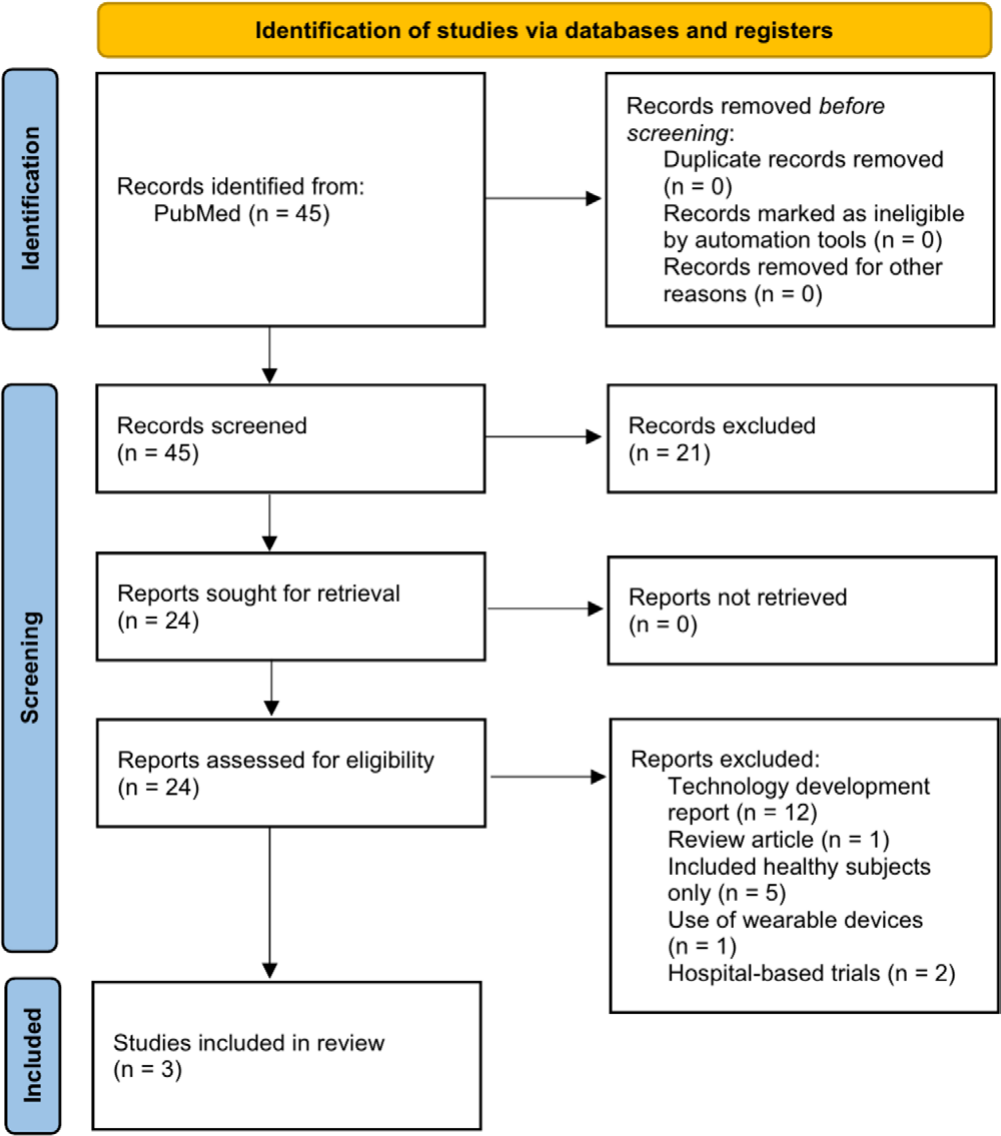
Flow chart for the selection of studies.

In total, 45 articles were identified, and all studies were exported to EndNote X9 for the evaluation of eligibility. After initially reviewing the titles, abstracts, and keywords of all the studies, 21 articles that did not align with the subject were excluded. Following a detailed examination of 24 full-text papers, 21 were excluded for the following reasons: 12 technical development reports, 1 review paper, 5 studies exclusively involved healthy participants, 1 used wearable devices, and 2 assessed the performance of noncontact sensors based on hospital environments (Fig. 1). Finally, 3 papers^[24–26]^ were selected, and the characteristics of each included study are described in Table 1.

**Table 1:** Characteristics of selected studies.

Study	Participants	Target disorder	Noncontact sensor	Conventional measurement methods	Outcome parameters
Ballal et al 2014^[24]^	N = 20 Mean age = 68 ± 5.9 years	Chronic obstructive pulmonary disease	Radio frequency biomotion sensor (SleepMinder™, ResMed Sensor Technologies, Dublin, Ireland)	Polysomnography	Respiratory rate
Crinion et al 2020^[25]^	Sleep clinic N = 67 Mean age = 52 ± 13 years Hypertension clinic N = 55 Mean age = 58 ± 9 years	Sleep apnea	Radio frequency biomotion sensor (SleepMinder™, ResMed Sensor Technologies, Dublin, Ireland)	Polysomnography and polygraphy	Apnea–hypopnea index
Harrington et al 2021^[26]^	Healthy volunteers N = 5 Mean age = no information Patient with heart failure N = 1 Mean age = 60 years	Ischemic cardiomyopathy	BedScales	Polysomnography and commercial scales	Respiratory rate, heart rate, and body weight

### 3.2 . Application of noncontact sensors in a home setting

**Figure 2.**
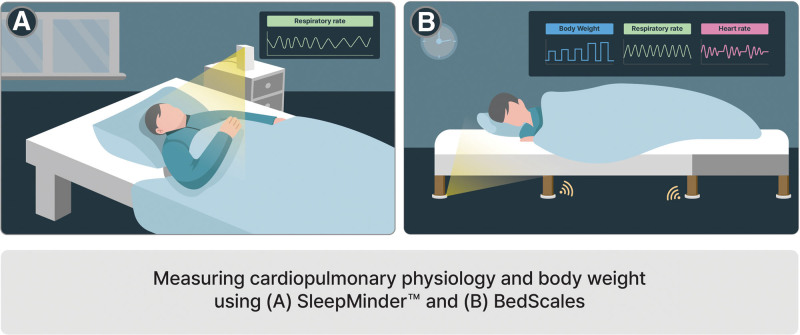
Summary illustration of this narrative review.

In 2014, Ballal et al^[24]^ conducted a study monitoring the respiratory movements of 20 patients with chronic obstructive pulmonary disease (COPD) over 8 weeks using noncontact sensors (SleepMinder™, ResMed Sensor Technologies, Dublin, Ireland) (Fig. 2). They investigated the association between the patterns of nighttime respiratory rates, analyzed using noncontact sensors, and the clinical condition of the participants. The participants, classified as Gold stages 2 to 4 (moderate to very severe), experienced exacerbation of COPD (ECOPD)-related hospital admissions within the 12 months preceding the study. Each patient was provided with a noncontact device called SleepMinder™ to collect respiratory information during sleep. An automatic algorithm called the adaptive notch filter (ANF) was applied to SleepMinder to analyze signals while the patient was lying in bed. Data were recorded on the built-in Secure Digital card of the device. The accuracy of the ANF was validated by comparison with stable-state respiratory signals extracted from polysomnography (PSG) during sleep studies. The root mean square error for respiratory rate was approximately 0.35 breath per minute, and the respiratory rate estimated within a margin of error of 1 was > 98%. This suggests that under quiet breathing conditions, noncontact sensors and the ANF algorithm can generate robust estimates of respiratory rates. Noncontact sensors can thus be effectively utilized to measure respiratory rates in a home environment. To achieve the ultimate goal of this study, the association between the patterns of analyzed nighttime respiratory rates with ANF and the occurrence of ECOPD was investigated. Furthermore, ECOPD was defined as hospitalization or irregular hospital visits. Further, if the respiratory rate per minute on a specific date was higher than the values of the previous 5 days, the following day was considered to potentially indicate an occurrence of ECOPD. In all, 12 potential ECOPD events were predicted, and 19 events occurred in 11 patients, resulting in a sensitivity of 63% and a specificity of 85%. Overall, predicting ECOPD occurrence based on an increased respiratory rate per minute is considered a valid approach.

In 2020, Crinion et al^[25]^ evaluated the performance of SleepMinder™ in estimating the severity of obstructive sleep apnea (OSA) (Fig. 2). The study targeted 122 patients suspected of having sleep apnea and installed SleepMinder™ in their homes for a 7-day monitoring period. Among the 122 participants, 67 were patients attending a sleep clinic with a clinical suspicion of sleep apnea, and the remaining 55 were individuals diagnosed with hypertension who were attending a hypertension clinic and were currently undergoing treatment for hypertension. All patients received and installed the SleepMinder™ in their homes, and after 7 days of monitoring, they were admitted to the hospital. The sleep clinic patients underwent PSG and patients with hypertension underwent polygraphy (PG). The accuracy of the device was evaluated by comparing the average apnea–hypopnea index (AHI) estimated using SleepMinder with that estimated using PSG or PG. The AHI is an assessment tool that calculates the average number of apneas and hypopneas per hour during the total sleep time. The threshold for defining OSA is set at AHI ≥ 15 (none or minimal, AHI < 5 per hour; mild, 5 ≤ AHI < 15 per hour; moderate, 15 ≤ AHI < 30 per hour; severe, AHI ≥ 30 per hour). The correlation coefficient of AHI values between SleepMinder™ and PSG in sleep clinic patients was 0.68, and that of AHI values between SleepMinder™ and PG in the hypertension clinic patients was 0.7. Furthermore, in the sleep clinic group, the sensitivity and specificity for the diagnostic threshold of AHI ≥ 15 for SleepMinder™ were 72% and 94%, respectively. In the hypertension clinic group, these values were 50% and 72%, respectively. These results indicate the effectiveness of noncontact sensors in confirming OSA, highlighting their potential for monitoring populations with suspected OSA in a home setting.

In 2021, Harrington et al^[26]^ validated the performance of the noncontact measurement device, “BedScales,” for measuring body weight, respiration, and heart rate by comparing it with commercial scales and PSG measurements (Fig. 2). BedScales is a noncontact device for measuring biometric information and comprises 4 individual pads. The device’s performance was evaluated during development using 5 healthy volunteers. Subsequently, the device was installed in the home of 1 patient with chronic disease for 3 months to confirm its durability. During the performance evaluation of BedScales targeting healthy individuals, noncontact sensors were individually installed under each of the 4 legs of the bed. For weight measurements, when the same load was applied at different positions, the 4 interconnected sensors were calibrated by adjusting the coefficients to minimize dispersion. The weight measured using BedScales was compared to that measured using commercial scales, resulting in a mean error of −0.057%. The respiratory signals measured using BedScales originated from the dynamic redistribution of the load with chest movements during both inspiration and expiration while asleep. The respiratory signal measured using BedScales was compared with the values simultaneously measured using a standard commercial chest belt respiratory monitor. The mean error ± standard deviation between BedScales and the chest belt for the respiratory signal was −0.17 ± 0.72 beats per minute (bpm). Heart rate was validated by generating a single peak estimate from the ballistocardiogram (BCG) signal produced with each heartbeat and comparing it with concurrently recorded electrocardiography values (mean error ± standard deviation = −0.94 ± 2.14 beats per minute). No significant differences were observed between the BedScales and standard testing tools in any of the measurement results for weight and cardiopulmonary physiology. Performance-validated BedScales were proven to be durable through actual use in the home (installed on a home recliner used every night during sleep) of a patient with heart failure and ischemic cardiomyopathy. The patient was monitored for weight and cardiopulmonary physiology using BedScales for 7 days before and 3 months after coronary artery bypass grafting. The measured parameters were automatically transmitted to a web cloud for data management, and the noncontact sensor device operated without issues in the home environment over an extended period. In conclusion, BedScales demonstrates a performance similar to that of existing weight and cardiopulmonary measurement devices and exhibits excellent durability for heavy bedroom furniture. BedScales are thus considered effective for the long-term home monitoring of patients with chronic diseases.

### 3.3 . Risk of bias

In all 3 studies^[24–26]^ included in this review, it was unlikely that the status and interpretation of the subjects defined by the reference standard or index test led to bias. However, as the trials were conducted in a home environment, situations beyond the control of medical staff (e.g., patient movement during sleep, changes in vital signs due to alcohol consumption) could have influenced the measurement results. Additionally, while 2 studies^[24,25]^ were determined to have enrolled consecutive patients within a same community, 1 study^[26]^ involved only a single patient, which means the patient selection might not represent the entire population with the same disease, potentially introducing bias. Overall, the risk of bias was judged to be low for the 3 included studies,^[24–26]^ but caution is needed when interpreting the results due to some domains, such as patient selection, patient flow, or blinding of assessment, having unclear or high risks of bias (Table 2).

**Table 2:** Quality assessment of included studies using the quality assessment of diagnostic accuracy studies (QUADAS)-2 tool.

Major components	Ballal et al 2014^[24]^	Crinion et al 2020^[25]^	Harrington et al 2021^[26]^
*Patient selection*			
1. Was a consecutive or random sample of patients enrolled?	Yes	Yes	No
2. Was a case–control design avoided?	Yes	Yes	Yes
3. Did the study avoid inappropriate exclusions?	Unclear	Unclear	Unclear
4. Could the selection of patients have introduced bias?	Low	Low	High
5. Are there concerns that the included patients do not match the review question?	Low	Low	Low
*Index test*			
6. Were the index test results interpreted without knowledge of the results of the reference standard?	Unclear	Yes	Unclear
7. If a threshold was used, was it pre-specified?	Yes	Yes	Yes
8. Could the conduct or interpretation of the index test have introduced bias?	Low	Low	Low
9. Are there concerns that the index test, its conduct, or interpretation differ from the review question?	Low	Low	Low
*Reference standard*			
10. Is the reference standard likely to correctly classify the target condition?	Yes	Yes	Yes
11. Were the reference standard results interpreted without knowledge of the results of the index test?	Unclear	Yes	Unclear
12. Could the reference standard, its conduct, or its interpretation have introduced bias?	Low	High	Low
13. Are there concerns that the target condition as defined by the reference standard does not match the review question?	Low	Low	Low
*Flow and timing*			
14. Was there an appropriate interval between index test(s) and reference standard?	Yes	Yes	Yes
15. Did all patients receive a reference standard?	Yes	Yes	Yes
16. Did all patients receive the same reference standard?	Yes	No	Yes
17. Were all patients included in the analysis?	Yes	Yes	Yes
18. Could the patient flow have introduced bias?	Unclear	High	Low

## 4. Discussion

In this study, 3 trials^[24–26]^ involving the installation of noncontact sensors in homes and the evaluation of their performance were reviewed. Two of these studies^[24,25]^ monitored respiratory status during sleep using SleepMinder™, while 1 study^[26]^ monitored weight and cardiorespiratory physiology using BedScales. According to these 3 studies,^[24–26]^ noncontact sensors can be effectively utilized for long-term monitoring of the health status of patients with sleep apnea or chronic conditions in a home setting.

SleepMinder™ is a device developed to measure the movements of a sleeping person and is primarily used to estimate the severity of OSA by analyzing respiratory patterns.^[21]^ The device operates by transmitting 2 short wireless frequency energy pulses, where the first pulse serves as the main transmission pulse and the second acts as a mixer pulse.^[24]^ The device operates by mixing the reflected pulse received by the sensor with the mixer pulse inside the receiver. This device is designed to generate a signal proportional to the phase change in the reflected pulse.^[24]^ SleepMinder™ is compact and easy to carry and is typically installed near the patient’s bed, ensuring it does not disturb sleep.^[27]^ In contrast, PSG, the gold standard test for diagnosing sleep disorders in clinical settings, involves inconveniences such as testing in unfamiliar environments and potential negative impacts on sleep quality owing to discomfort caused by the equipment.^[28]^ SleepMinder™ is considered capable of sufficiently addressing the drawbacks of PSG. However, because of its directional nature, SleepMinder™ can only measure the forward movement of the sensor and has a range limit that responds only to objects within 1.5 meters.^[24]^ Additionally, the device is usually installed at a position higher than that of a lying patient, potentially causing inconvenience in securing the space and furniture that meet the height conditions by the bedside. Moreover, further research is needed to enhance the device’s performance. In the study by Ballal et al,^[24]^ the sensitivity of SleepMinder™ in predicting ECOPD was 63% whereas the sensitivity for estimating OSA severity in the study by Crinion et al^[25]^ ranged from 50% to 72%. SleepMinder™ can be influenced by various potential factors that can negatively impact the extraction and analysis of respiratory signals, such as sleeping positions, including prone or side-lying positions, movements during sleep, or deterioration of sleep quality because of alcohol consumption. Uncontrollable and unpredictable factors in a home environment can affect data processing because medical staff cannot control these factors. Notably, since limb movements during sleep can lead to an overestimation of the AHI, employing the novel sleep disorder index, which combines AHI and the periodic limb movement Index, allows for a more accurate detection of sleep-disordered breathing. In 2018, Weinreich et al^[27]^ reported that the use of sleep disorder index enabled SleepMinder™ to accurately detect the combination of sleep-disordered breathing and periodic limb movement with a sensitivity of 92.2% and a specificity of 95.8%. Further, Crinion et al^[25]^ demonstrated a significant difference in sensitivity for estimating OSA severity between groups from sleep and hypertension clinics (72% for the sleep clinic group and 50% for the hypertension clinic group). This was considered to arise from the characteristics of the groups, with the sleep clinic group comprising patients with OSA symptoms, and the hypertension clinic group comprising patients without OSA.^[25]^ These results indicate that SleepMinder™ performs better in targeting patients with severe OSA and emphasizes the necessity for technological advancements to generate accurate AHI scores in individuals with or without mild OSA symptoms. Additionally, since SleepMinder™ is a radar-based noncontact sensor, it is necessary to address the technical limitations associated with radar sensors. To prevent signal distortion or reduced accuracy due to interference from other electronic devices, it may be beneficial to develop more sophisticated signal processing and filtering algorithms.^[29]^

BedScales is an under-the-bed mechanical sensing platform developed for noncontact physiological monitoring.^[26]^ The sensor of the device is attached to the bed or sofa with legs that the patient primarily uses, and all data are communicated through Wi-Fi and transmitted to a web cloud. The weight is calculated by comparing the total load measured before and after adding the target weight to the bed.^[26]^ Cardiopulmonary signals are designed to be generated using frequency-dependent filtering with cutoffs of 0.167 Hz and 1.5 Hz for breathing signals and 5 Hz and 50 Hz for BCG signals.^[26]^ Additionally, an algorithm was incorporated to separate weight and respiratory signals, even when sharing the bed with a partner or pet.^[26]^ However, the technology to separate mixed BCG signals from more than 2 individuals has not yet been implemented and remains a challenge to address in the future. To separate mixed signals from 2 or more individuals, techniques such as independent component analysis, principal component analysis, waveform analysis and time series analysis, or Bayesian separation techniques can be considered.^[30,31]^ In a study conducted by Harrington et al,^[26]^ BedScales proved its potential to monitor the health status of patients with chronic cardiopulmonary diseases, identify pre-admission signs, and improve the treatment environment. However, a critical limitation is its incompatibility with furniture lacking legs or that fixed to the floor. Therefore, incorporating mattress-type sensor that detects biometric signals through pressure changes might be more advantageous for commercialization.^[32,33]^ Furthermore, since the study was conducted with only 1 patient, it is impractical to generalize the findings to a population with the same condition. Hence, future studies should focus on validating the device’s performance in home settings with a larger patient group.

Previously, home health monitoring usually relied on wearable devices or required users to perform self-measurements.^[34]^ This frequently presented challenges for patients unfamiliar with the devices as it demanded proficiency in handling machinery and transmitting data through applications.^[14]^ In contrast, the noncontact sensor devices introduced in this review automatically extracted and analyzed data once installed, minimizing the burden on users. Furthermore, because these devices do not disturb the patient during sleep, they are expected to facilitate the easy monitoring of health conditions during sleep in home settings without compromising sleep quality. However, it is important to note that the technologies developed so far have primarily been tested and validated in situations with minimal movement during sleep. Therefore, these devices are considered useful mainly during sleep. In actual home environments, daily activities encompass movements beyond sleep, requiring the development of technologies capable of monitoring health status in areas outside the bedroom. Researching the implementation of an enhanced disease management system compared with the current state is thus important.

## Author contributions

**Conceptualization:** Yoo Jin Choo, Gun Woo Lee, Jun Sung Moon, Wook-Tae Park, Heeyeon Won, Min Cheol Chang.

**Data curation:** Yoo Jin Choo, Gun Woo Lee, Jun Sung Moon, Wook-Tae Park, Heeyeon Won, Min Cheol Chang.

**Formal analysis:** Yoo Jin Choo, Gun Woo Lee, Jun Sung Moon, Wook-Tae Park, Heeyeon Won, Min Cheol Chang.

**Funding acquisition:** Yoo Jin Choo, Gun Woo Lee, Jun Sung Moon, Wook-Tae Park, Heeyeon Won, Min Cheol Chang.

**Investigation:** Yoo Jin Choo, Gun Woo Lee, Jun Sung Moon, Wook-Tae Park, Heeyeon Won, Min Cheol Chang.

**Methodology:** Yoo Jin Choo, Gun Woo Lee, Jun Sung Moon, Wook-Tae Park, Heeyeon Won, Min Cheol Chang.

**Resources:** Yoo Jin Choo, Gun Woo Lee, Jun Sung Moon, Wook-Tae Park, Heeyeon Won, Min Cheol Chang.

**Software:** Yoo Jin Choo, Gun Woo Lee, Jun Sung Moon, Wook-Tae Park, Heeyeon Won, Min Cheol Chang.

**Supervision:** Gun Woo Lee, Jun Sung Moon, Min Cheol Chang.

**Validation:** Yoo Jin Choo, Gun Woo Lee, Jun Sung Moon, Wook-Tae Park, Heeyeon Won, Min Cheol Chang.

**Visualization:** Yoo Jin Choo, Gun Woo Lee, Jun Sung Moon, Wook-Tae Park, Heeyeon Won, Min Cheol Chang.

**Writing – original draft:** Yoo Jin Choo, Gun Woo Lee, Jun Sung Moon, Wook-Tae Park, Heeyeon Won, Min Cheol Chang.

**Writing – review & editing:** Yoo Jin Choo, Gun Woo Lee, Jun Sung Moon, Wook-Tae Park, Heeyeon Won, Min Cheol Chang.

## Supplementary Material


